# *Orthosiphon stamineus* Leaf Extract Affects TNF-α and Seizures in a Zebrafish Model

**DOI:** 10.3389/fphar.2018.00139

**Published:** 2018-02-23

**Authors:** Brandon Kar Meng Choo, Uday P. Kundap, Yatinesh Kumari, Seow-Mun Hue, Iekhsan Othman, Mohd Farooq Shaikh

**Affiliations:** ^1^Neuropharmacology Research Laboratory, Jeffrey Cheah School of Medicine and Health Sciences, Monash University Malaysia, Bandar Sunway, Malaysia; ^2^School of Science, Monash University Malaysia, Bandar Sunway, Malaysia

**Keywords:** epilepsy, *Orthosiphon stamineus*, zebrafish model, TNF-α, anticonvulsant activity

## Abstract

Epileptic seizures result from abnormal brain activity and can affect motor, autonomic and sensory function; as well as, memory, cognition, behavior, or emotional state. Effective anti-epileptic drugs (AEDs) are available but have tolerability issues due to their side effects. The Malaysian herb *Orthosiphon stamineus*, is a traditional epilepsy remedy and possesses anti-inflammatory, anti-oxidant and free-radical scavenging abilities, all of which are known to protect against seizures. This experiment thus aimed to explore if an ethanolic leaf extract of *O. stamineus* has the potential to be a novel symptomatic treatment for epileptic seizures in a zebrafish model; and the effects of the extract on the expression levels of several genes in the zebrafish brain which are associated with seizures. The results of this study indicate that *O. stamineus* has the potential to be a novel symptomatic treatment for epileptic seizures as it is pharmacologically active against seizures in a zebrafish model. The anti-convulsive effect of this extract is also comparable to that of diazepam at higher doses and can surpass diazepam in certain cases. Treatment with the extract also counteracts the upregulation of NF-κB, NPY and TNF-α as a result of a Pentylenetetrazol (PTZ) treated seizure. The anti-convulsive action for this extract could be at least partially due to its downregulation of TNF-α. Future work could include the discovery of the active anti-convulsive compound, as well as determine if the extract does not cause cognitive impairment in zebrafish.

## Introduction

Epileptic seizures are typically described as a short-term manifestation of numerous signs and/or symptoms because of unusually superfluous or concurrent activity in the brain. In contrast, epilepsy is a collection of neurological disorders characterized by the lasting tendency to spawn epileptic seizures (Fisher et al., 2014). Epilepsy is a serious disorder of the Central Nervous System (CNS) as the global epilepsy prevalence is approximately one in 100 people according to Holland (2014). Whilst the underlying cause of epilepsy is not always clear, anti-convulsant drugs or anti-epileptic drugs (AEDs) as they are commonly known, may be used for the symptomatic treatment of epilepsy. The older generation of AEDs have side effects which range from abdominal discomfort and anorexia to psychosis and aplastic anemia; together with an array of different idiosyncratic reactions. In comparison, AEDs from the newer generation can result in side effects which range from fatigue and drowsiness to vomiting and diplopia (French and Gazzola, 2011). Whilst the efficacy of the AEDs in use today has been demonstrated, a need for the discovery of new AEDs with fewer side effects remains.

*Orthosiphon stamineus* is a Malaysian herb also known locally as ‘misai kucing’ and is widely grown in tropical regions which have high temperatures and year-round rainfall (Akowuah et al., 2004). In the Southeast Asian region, *O. stamineus* leaves are harvested and dried to make tea leaves (Gan et al., 2017). The *O. stamineus* tea leaves can then be brewed into a herbal tea and used as a traditional medicine to treat epilepsy (Hossain and Mizanur Rahman, 2015). An extract of *O. stamineus* leaves has been found to possess anti-inflammatory, (Yam et al., 2008) anti-oxidant and free-radical scavenging abilities (Yam et al., 2007). Although the exact mechanism leading to the formation of seizures is unknown, there is evidence that pro-inflammatory mediators released by the brain and peripheral immune cells play a role (Vezzani et al., 2011). There has also been an indication that oxidative stress has a role in epilepsy, given the high degree of oxidative metabolism, limited antioxidant defense and the abundance of polyunsaturated fatty acids in the brain. It is possible that these conditions increase the vulnerability of the brain to free radical damage, leading to certain types of epilepsy (Devi et al., 2008). An experiment by Yam et al. (2010) suggested that the components of *O. stamineus* leaves which are responsible for its anti-inflammatory effect in a chloroform extract are the polymethoxylated flavones sinensetin, eupatorine and 30-hydroxy-5,6,7,40-tetramethoxyflavone; which possibly function by inhibiting the nitric oxide pathway and the synthesis of prostaglandin. Akowuah et al. (2005) also found that sinensetin, eupatorine, 30-hydroxy-5,6,7,40-tetramethoxyflavone, rosmarinic acid and quercetin form the major components in an *O. stamineus* extract which possess significant free radical scavenging and antioxidant ability. Thus, the properties of *O. stamineus* combined with its traditional usage for the treatment of epilepsy makes it an encouraging candidate for the development of novel AEDs.

One of the most frequently used approaches to inducing seizures in animals are chemoconvulsants. An example of a chemoconvulsant among many the different available is pentylenetetrazol (PTZ). PTZ is believed to induce seizures primarily by binding to the γ-Aminobutyric Acid (GABA_A_) receptor and impeding the neuroinhibitory action of GABA (Berghmans et al., 2007). Although the majority of past research concerning epilepsy has been undertaken using rodents as the animal model, zebrafish are currently becoming increasingly popular as a model for epilepsy. One possible reason for this is that dissolving the compounds to be tested directly in the zebrafish tank water becomes an option, which eliminates the necessity of performing an invasive procedure such as an injection. Despite zebrafish being fish and hence more removed from humans in an evolutionary perspective in comparison to the mammalian rodents, their genes are nevertheless still around 75% homologous to human genes (Baraban et al., 2005; Berghmans et al., 2007). Among the other aspects in which zebrafish are superior to rodents as an animal model are their longer lifespan and robust phenotypes, as they display obvious and easily quantifiable behavioral endpoints (Stewart et al., 2012). The blood brain barrier in zebrafish is also tight-junction based, and highly permeable to macromolecules, meaning that zebrafish will be extremely responsive the compounds being tested (Eliceiri et al., 2011). It is for these reasons that this experiment utilized zebrafish as an animal model of epilepsy over rodents.

Once the animal model of epilepsy and the method of inducing seizures is ascertained, a technique for assessing compounds believed to be anti-convulsive is needed. One way of doing this is to test adult zebrafish inside a tank in which they can be observed so that their seizure behavior can be scored according to a predefined scoring system. Both the top and side points of view for the observation tank can be utilized for the neurophenotypic classification of the responses which result in chemoconvulsant treated adult zebrafish, as they are very similar to those observed during a seizure. Whilst the abnormal response displayed by the zebrafish varies based on the chemoconvulsant used, the conventional endpoints which are used include rapid twitching, loss of body posture, hyperactive, spiral or circular swimming, paralysis or immobility, body contractions similar to spasms and death (Stewart et al., 2012). An induced seizure has also been shown using rodent models of epilepsy, to also result in an upregulation of specific genes at the site where the seizure was initiated. The upregulated genes are known as immediate-early genes (Morgan et al., 1987) and are comprised of genes such as the early proto-oncogene c-Fos, which also functions as a neuronal activation marker. A similar pattern in the upregulation of seizure related genes during an induced seizure is also present in zebrafish brains (Stewart et al., 2012) and may be quantitively examined to possibly serve as biomarkers of brain disorders.

Thus, whilst the efficacy of the AEDs used today for the symptomatic treatment of epilepsy is proven, there is still a necessity for the discovery of new AEDs with comparable efficacies, but with fewer side effects. Given its beneficial properties and traditional usage, *O. stamineus* leaves have the potential to be a novel treatment for epilepsy. Thus, this study aimed to determine if an ethanolic leaf extract of *O. stamineus* is pharmacologically active against seizures. This was done by observing whether pre-treating zebrafish with varying doses of the extract has any effect on the progression of PTZ-induced seizures. This experiment involved the use of three different treatment doses of the *O. stamineus* ethanolic leaf extract; with the exact concentrations decided based on a prior toxicity study using adult zebrafish. The last part of this study involved harvesting the zebrafish brains for gene expression studies to help determine the mechanism of action by which an ethanolic leaf extract of *O. stamineus* exerts its anti-convulsive effect in the zebrafish brain as the expression level of certain genes changes characteristically after a seizure (Morgan et al., 1987).

## Materials and Methods

### Materials

#### Chemicals

The standardized *O. stamineus* ethanolic leaf extract was purchased from Natureceuticals Sdn Bhd. According to the manufacturer, the extract was a 50% ethanolic extract prepared using Digimaz technology. Pentylenetetrazol (PTZ) and the standard AED diazepam (DZP) were purchased from Sigma–Aldrich (United States). TRIzol^®^ reagent was purchased from Invitrogen, Carlsbad, CA, United States. For the gene expression study, QuantiTect SYBR Green dye (Qiagen, Valencia, CA, United States) was used together with the following primer sets:

BDNF: Dr_bdnf_1_SG QuantiTect Primer Assay (Cat no. QT02125326);

NF-κB: Dr_nfkb1_2_SG QuantiTect Primer Assay (Cat no. QT02498762);

NPY: Dr_npy_1_SG QuantiTect Primer Assay (Cat no. QT02205763);

c-Fos: Dr_fos_1_SG QuantiTect Primer Assay (Cat no. QT02103243);

TNF-α: Dr_tnf_1_SG QuantiTect Primer Assay (Cat no. QT02097655);

IL-1: Dr_il1rapl1a_1_SG QuantiTect Primer Assay (Cat no. QT02131850);

eef1a1b: Dr_eef1a1b_2_SG QuantiTect Primer Assay (Cat no. QT02042684)

#### Software and Equipment

The Smart V3.0.05 tracking software (Pan Lab, Harvard apparatus) was used for the automated tracking of zebrafish swimming patterns. The Applied Biosystems StepOnePlus^TM^ Real-Time PCR System was used for the gene expression study.

#### Animals

Adult zebrafish (*Danio rerio*) 3–4 months of age and of the heterogeneous wild-type strain with a typical short-fin phenotype were purchased at the aquarium shop ‘Akarium Batu Karang Laut’ (Subang Jaya, Malaysia). All zebrafish were held at the Monash University Malaysia animal facility under standard husbandry conditions. The zebrafish tanks were kept at a water temperature of between 26 and 30°C, a water pH of between pH 6.8 and pH 7.1 and under a 250-lux light intensity with a cycle of 14-h of light to 10 h of darkness. The lights were automatically turned on at 8 am and automatically turned off at 10 pm via a timer. The zebrafish were fed thrice a day with TetraMin^®^ Tropical Flakes and their diet was supplemented with live brine shrimps (*Artemia*) purchased from Bio-Marine (Aquafauna, Inc. United States). Standard zebrafish tanks with a length of 36 cm, a width of 26 cm and a height of 22 cm were used to house the zebrafish. The tanks were equipped with a water circulation system to provide constant aeration. Group housing, whereby 10–12 fish were kept per tank, was practiced with the females and males being housed separately. All animal experimentation was authorized by the Monash Animal Research Platform (MARP), Australia.

### Methods

#### Toxicity Study

A zebrafish toxicity study was carried out on adult zebrafish to determine the exact *O. stamineus* ethanolic leaf extract concentrations to be used with each of the three treatment groups. A limit test was first performed based on a modified version of the OECD Guidelines for the Testing of Chemicals No. 203 (OECD, 1992). An observation tank was first set up and filled with 13 L of the water normally used to fill the zebrafish tanks. One zebrafish from the untreated normal control group was then placed in the tank and its behavior was recorded for 10 min. After each recording, the zebrafish was transferred into individual 1 L tanks filled with the same water. The procedure then was repeated for each of the seven zebrafish in the control group. The recording and tank transfer procedure was then repeated with the seven zebrafish of the treatment group but with the extract added to the water to make up a concentration of 100 mg/L. All 14 zebrafish were then kept for 96 h in their respective one-liter tanks. All 14 zebrafish were checked on every 15 min for the first 2 h of exposure and every half an hour thereafter for the first day. On subsequent days, the zebrafish were checked on thrice daily during the morning, afternoon and evening. Any zebrafish found to exhibit severe symptoms of pain or suffering according to our predefined monitoring sheet at any checkpoint were humanely euthanized via an overdose of benzocaine. If no zebrafish require euthanasia at the limit concentration, the extract concentration will be raised by a factor of 2–200 mg/L and the test protocol repeated. If there is at least one zebrafish requiring euthanasia at the limit concentration, the concentration will be decreased by a factor of two, to 50 mg/L and the test protocol repeated. This protocol deviates from the OECD guidelines in that it does not use mortality as the criterion to determine toxicity due to the concerns of the MARP-Australia in using death as an endpoint. The highest dose which did not require euthanasia of any zebrafish was used as the ‘High’ dose in the following behavioral study with the ‘Medium’ dose and ‘Low’ dose being a factor of two and four lower than the ‘High’ dose, respectively.

#### Behavioral Study

##### Drug treatment and groups

Three-month-old adult zebrafish with weights ranging from 0.4 to 0.8 g were selected. The zebrafish were then divided into six groups, with 10 fish per group. PTZ was dissolved in distilled water whereas DZP and the *O. stamineus* extract was dissolved in the same water used to fill the zebrafish tanks.

Group I: Vehicle Control (CP), Tank Water Only;

Group II: Negative Control (CN), PTZ (170 mg/kg) Only;

Group III: Positive Control (CP), DZP (10 mg/L) + PTZ (170 mg/kg);

Group IV: Treatment Group 1, *O. stamineus* extract (Low dose) + PTZ (170 mg/kg);

Group V: Treatment Group 2, *O. stamineus* extract (Medium dose) + PTZ (170 mg/kg);

Group VI: Treatment Group 3, *O. stamineus* extract (High dose) + PTZ (170 mg/kg)

##### Procedure for a zebrafish intraperitoneal injection

All intraperitoneal injections were administered into the abdominal cavity at a location posterior to the pelvic girdle, using a 10 μl Hamilton syringe (700 series, Hamilton 80400) (Stewart et al., 2011). The experiment was performed in a separate behavior room with the room temperature kept between 26 and 30°C and humidity between 50 and 60%. All zebrafish were acclimatized in the said behavior room for 2 hours prior to experiment for the purpose of minimizing any novel tank response. Other precautions taken include using a small injection volume of 10 μl per gram of fish and using a 35-gage needle. The zebrafish were restrained in a water saturated sponge under benzocaine anesthesia to reduce the distress inflicted on the zebrafish (Júnior et al., 2012). This intraperitoneal injection technique was found to be effective in zebrafish (Kundap et al., 2017) and did not cause any mortality throughout the experiment.

Each zebrafish was captured individually using a fish holding net, and then transferred into an anesthesia solution (30 mg/L Benzocaine). The zebrafish was taken out once anesthetized and then weighed to calculate the dose and hence the injection volume. A soft sponge approximately 20 mm in height was saturated with water and set inside a 60 mm Petri dish. A cut between 10 and 15 mm in depth was made in the sponge to restrain and hold the fish for the intraperitoneal injection. The intraperitoneal injection was given while using a dissecting microscope by inserting the needle into the midline between the pelvic fins. An appropriate volume was then injected into the zebrafish, after taking into account the body weight of the zebrafish. After the intraperitoneal injection, the zebrafish was immediately transferred to an observation tank.

##### PTZ-Induced Seizure Model

The zebrafish were habituated for 30 min in 1 L treatment tanks filled with 1 L of the water normally used to fill the zebrafish tanks, before administration of PTZ. Groups I and II were habituated in tanks only containing the water normally used to fill the zebrafish tanks. Groups III to VI had either diazepam (10 mg/L) or the extract added to the tank water. After the 30-min habituation time, the zebrafish from groups II to VI were injected with PTZ (170 mg/kg, IP). Group I zebrafish did not receive any injections. PTZ injected zebrafish present diverse seizure profiles, intensities and latency in reaching the different seizure scores. PTZ-induced seizures will persist for about 10 min after the PTZ injection and gradually decrease with time. The PTZ injected adult zebrafish were then moved to a 13-L observation tank filled three quarters of the way with water. The behavior of the zebrafish was then recorded for 10 min after recovery from anesthesia and the video was later viewed using computer to determine the highest seizure score every minute. The zebrafish seizure score was recorded as per the scoring system used by Kundap et al. (2017) and is given below.

Score 1 - Short swim mainly at the bottom of the tank

Score 2 - Increased swimming activity and high frequency of opercular movement

Score 3 - Burst swimming, left and right movements as well as erratic movements

Score 4 - Circular movements

Under the directives of MARP-Australia, the PTZ dose was set at 170 mg per kg of zebrafish body weight in order to limit the resulting seizure scores to a maximum of four. Time to score four seizure onset (seconds) and mean seizure score over 10 min were noted when viewing the recorded video. The mean seizure score over 10 min was calculated by first assigning the highest observed seizure score 1 min after the start of the video, as the seizure score for the first minute. This process was repeated until the end of the 10th min and all 10 seizure scores were averaged to obtain the mean seizure score over 10 min. The zebrafish swimming pattern was determined via analysis using the Smart tracking software. The dose of PTZ (170 mg/kg) and the duration of the behavior recording (10 min) represent the standard protocol of our laboratory for inducing seizures with PTZ, as determined previously by Kundap et al. (2017). The diazepam dose (10 mg/L) and the habituation time (30 min) were chosen based on the results of an unpublished preliminary trial using the same methodology. The diazepam dose and the habituation time were varied till a mean seizure score over 10 min of less than one was obtained.

#### Gene Expression Study

##### Brain harvesting

After the behavioral study, the zebrafish brains were harvested by removing the zebrafish skull and extracting the brain, before transferring it straight into 200 μl of ice-cold TRIzol^®^. The zebrafish brains were then immediately stored at -80°C till required.

##### RNA isolation and synthesis of first strand cDNA

The mRNA was isolated according to the protocol supplied by the kit’s manufacturer, and was identical to the protocol used by Kundap et al. (2017). In short, the zebrafish brain was first homogenized whilst in TRIzol^®^ before chloroform was mixed in. The resulting mixture was then centrifuged at a speed of 13,500 rpm (revolutions per minute) for a period 15 min and at a temperature of 4°C. After centrifugation, the resulting aqueous supernatant was then transferred into a new tube before the addition of isopropanol. After mixing, the new tube was incubated for 10 min at room temperature and subsequently centrifuged for a period of 10 min at a speed of 13,500 rpm and at a temperature of 4°C. The resulting supernatant was removed and the pellet was rinsed with 75% ethanol. The pellet was then allowed to air dry for between 5 and 10 min. Nuclease-free water was then added to the tube for the purpose of dissolving the mRNA pellet. The purity and concentration of the resulting isolated mRNA was then measured with a NanoDrop Spectrophotometer. Afterwards, the isolated mRNA was then converted to cDNA as per the instructions given in the Omniscript Reverse-transcription Kit from QIAGEN.

##### StepOne^®^ real-time PCR

The gene expression level of Brain-Derived Neurotrophic Factor (BDNF), Nuclear Factor Kappa-light-chain-enhancer of activated B cells (NF-κB), Neuropeptide Y (NPY), c-Fos, Tumor Necrosis Factor alpha (TNF-α), Interleukin-1 (IL-1) and the housekeeping gene Elongation factor 1-alpha-1b (eef1a1b) were calculated via real-time quantitative RT-PCR (Applied Biosystems) together with QuantiTect SYBR Green dye and the appropriate Qiagen primer set for each gene; using a similar protocol to that used by Kundap et al. (2017). The samples were first incubated at 95°C for 2 min prior to thermal cycling. The thermal cycling settings used were 40 cycles of 95°C for 5 s, followed by 60°C for 15 s. The relative expression level (Fold Change) of the six genes of interest was calculated by normalizing the threshold cycle (Ct) values obtained from the genes of interest, against the Ct value of the eef1a1b housekeeping gene using the formula: 2 ^∧^ [Ct eef1a1b – Ct Gene of interest].

#### Statistical Analysis

All results were expressed as Mean ± Standard Error of the Mean (SEM). The data was analysed using one-way Analysis of Variance (ANOVA) and followed with Dunnett’s test. The PTZ only negative control group (Group II/CN) was used as the control for Dunnett’s test and all other groups were compared to it. The *P*-value, ^∗∗∗^*P* < 0.001 was regarded as statistically significant for the behavioral study, whereas a *P*-value of ^∗∗^*P* < 0.01 and ^∗^*P* < 0.05 was regarded as statistically significant for the gene expression study.

## Results

### Toxicity Study

**FIGURE 1:**
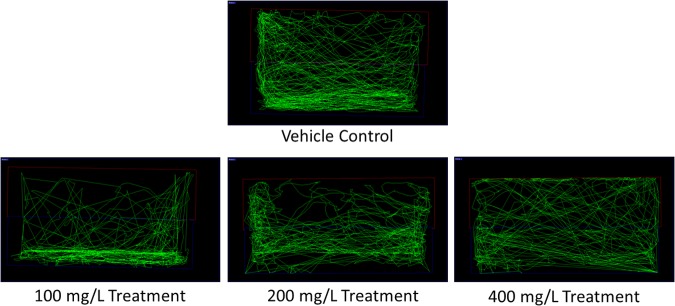
Representative zebrafish swimming pattern of the vehicle control and extract treated groups for the toxicity study.

The limit test performed using 100 mg/L of *O. stamineus* ethanolic leaf extract did not result in any mortality, morbidity or abnormal behavior in the zebrafish (*n* = 7). As per the protocol, the toxicity study was repeated using twice the concentration of the extract (200 mg/L) and again resulted in no mortality, morbidity or abnormal behavior in the zebrafish. Doubling the extract concentration once again to 400 mg/L produced no abnormal behavior in the zebrafish during the initial observation period, but later resulted in the death of all the treated zebrafish after an overnight exposure (less than 18 h after the last observation); during which the zebrafish were not monitored. From the results of the toxicity study, 200 mg/L was chosen to be the ‘High’ dose (T200) for the following behavioral study. The ‘Medium’ and ‘Low’ doses were thus chosen to be 100 mg/L (T100) and 50 mg/L (T50), respectively. From the software generated zebrafish swimming patterns (**Figure 1**), it was found that zebrafish treated with 100 mg/L of the extract spent more time at the bottom of the tank. This is in comparison to the vehicle control group (CV), which displayed a slight preference for the bottom of the tank but otherwise swam throughout the whole tank. In contrast, the zebrafish treated with 200 and 400 mg/L of the extract displayed no preference for any one location in the tank.

### Behavioral Study

#### Seizure Onset Time and Seizure Score Analysis

**FIGURE 2:**
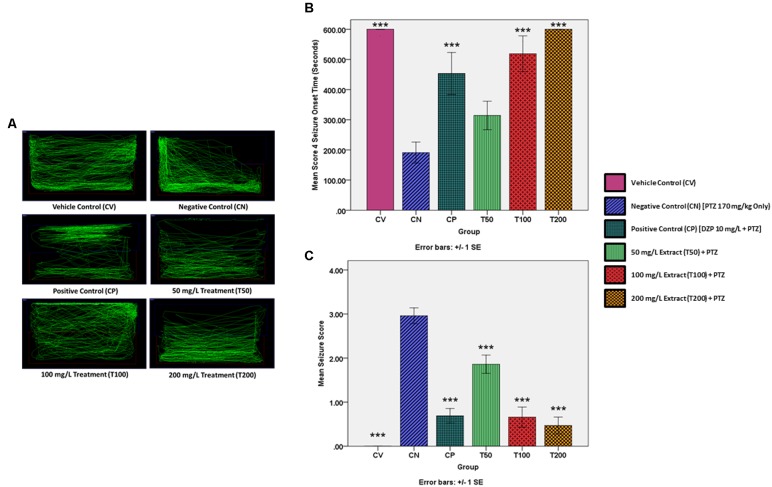
PTZ-induced behavior and locomotion for each experimental group. **(A)** Representative zebrafish locomotion pattern for the vehicle, negative and positive control groups, as well as the extract treated groups. **(B)** Represents the score 4 seizure onset time (seconds) for the vehicle and positive controls as well as the extract treated groups, as compared to the negative control (CN, 170 mg/kg PTZ Only) **(C)** Represents the mean seizure score for the vehicle and positive controls as well as the extract treated groups, as compared to the negative control (CN, 170 mg/kg PTZ Only). The data is expressed as Mean ± SEM, *n* = 10 and was analyzed using one-way ANOVA, followed with Dunnett’s test at significance level of ^∗∗∗^*P* < 0.001.

Mean seizure onset time for the untreated CV group was taken to be 600 s, or the entire length of the video and a mean seizure score of 0 was assigned to the untreated CV group. This is because the vehicle control zebrafish were not injected with PTZ and thus did not develop seizures. Injecting PTZ into the zebrafish in the CV group resulted in a significant decrease in mean seizure onset time to 191 s and a significant increase in mean seizure score to 2.96 in comparison to the CV group. The results of the CN group were then used as a baseline for the positive control and treatment groups. Pre-treating the zebrafish with the positive control drug diazepam (CP) before challenging them with PTZ, significantly increased the mean seizure onset time to 453.4 s and significantly reduced the mean seizure score to 0.69. In contrast, pre-treating the zebrafish with 50 mg/L of *O. stamineus* ethanolic leaf extract (T50 Group) increased the mean seizure onset time to 314.4 s, although this was statistically insignificant (*P* = 0.233). However, the decrease in mean seizure score of the T50 group to 1.86 was considered statistically significant. Doubling the extract pre-treatment dose to 100 mg/L (T100 Group) produced a significant increase in the mean seizure onset time to 518.8 s and a significant decrease in the mean seizure score to 0.66. The final treatment group (T200 Group) was pre-treated with 200 mg/L of extract and did not reach seizure score 4 and thus the mean seizure onset time was recorded as 600 s or the full length of the recorded video. The T200 group also had a significant decrease in the mean seizure score to 0.47. All results were considered significant at the significance level of ^∗∗∗^α = 0.001. The mean seizure onset time (seconds) and seizure score for each zebrafish group are presented in a graphical format in **Figure 2**.

#### Representative Locomotion Patterns

Using the Smart tracking software for the automated tracking of zebrafish swimming patterns, one representative swimming pattern was chosen for each group from among the *n* = 10 zebrafish per group. The representative swimming patterns are given in **Figure 2**. The normal zebrafish swimming behavior demonstrated by the zebrafish in the CV group is to spend roughly an equal amount of time swimming throughout the entire tank. In contrast, the untreated negative control zebrafish had a more erratic swimming pattern after the PTZ challenge, with the zebrafish dwelling at bottom of the tank more frequently. Pre-treatment with the standard AED diazepam modified the post PTZ challenge swimming behavior into a zig-zag like swimming pattern, with a significant amount of time being spent at the top and bottom of the tank. Pre-treatment with all three *O. stamineus* ethanolic leaf extract doses produced a swimming pattern similar to that of the normal control, although the 50 and 200 mg/L doses produced more bottom dwelling in the zebrafish. In comparison, the 100 mg/L dose produced the most similar swimming pattern to the vehicle control, but showed an increase in time spent at the water surface.

### Gene Expression Study

#### BDNF

**FIGURE 3:**
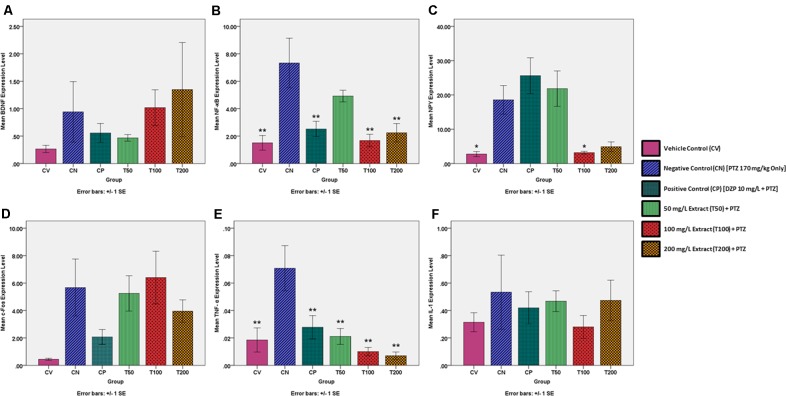
Expression levels of several genes in the brains of each experimental group as determined by real time-PCR. All changes in expression levels were as compared to the negative control group (CN, 170 mg/kg PTZ Only), and included the genes: **(A)** BDNF **(B)** NF-κB **(C)** NPY **(D)** c-Fos **(E)** TNF-α and **(F)** IL-1. The data is expressed as Mean ± SEM, *n* = 6 and was analyzed using one-way ANOVA, followed with Dunnett’s test at significance level of ^∗∗^*P* < 0.01 and ^∗^*P* < 0.05.

The change in the gene expression level of BDNF was determined to be statistically insignificant in all groups in comparison to the negative control at a level of ^∗^α = 0.05. However, when graphically represented in **Figure 3**, an increase BDNF expression by the CN group as compared to the CV group is visible. The BDNF expression level was reduced in both the CP and T50 groups, whereas the T100 and T200 produced an increase in BDNF expression level in comparison to the CN group.

#### NF-κB

There was a significant rise in the gene expression level of NF-κB for the CN group in comparison to the vehicle control (^∗∗^*P* < 0.01). The CP, T100 and T200 groups had a significant reduction in NF-κB expression (^∗∗^*P* < 0.01) as compared to the CN group. The T50 group also showed a reduction in NF-κB expression, but this did not approach statistical significance (*P* = 0.317). The NF-κB expression level for each zebrafish group is graphically represented in **Figure 3**.

#### NPY

There was a significant rise in NPY expression for the CN group in comparison to the vehicle control. In comparison to the negative control, only the T100 group showed a significant decrease in NPY expression. The CP, T50, and T200 groups did show a decrease in NPY expression but this was not significant at the level of ^∗^α = 0.05. The NPY expression level for each zebrafish group is graphically represented in **Figure 3**.

#### c-Fos

The change in the gene expression level of c-Fos was determined to be statistically insignificant in all groups in comparison to the negative control. However, when graphically represented in **Figure 3**, it can be seen that there is an increase c-Fos expression by the CN group as compared to the CV group. The level of c-Fos expression was decreased in the CP, T50, and T200 groups, whereas the T100 group had a decrease in c-Fos expression level when compared to the CN group.

#### TNF-α

There was a significant rise in the expression of TNF-α for the CN group as compared to the CV group. The CP, T50, T100, and T200 groups showed a significant reduction in the expression of TNF-α in comparison to the CN group. All changes in TNF-α expression were significant at the level of ^∗∗^α = 0.01. The TNF-α expression level for each zebrafish group is graphically represented in **Figure 3**.

#### IL-1

The change in the gene expression level of IL-1 was deemed to be statistically insignificant in all groups as compared to the negative control. However, when graphically represented in **Figure 3**, an increase IL-1 expression by the CN group as compared to the CV group is visible. The IL-1 expression level was reduced in the CP, T50, T100, and T200 groups as compared to the CN group. The IL-1 expression level for each zebrafish group is graphically represented in **Figure 3**.

## Discussion

This work aims to determine if an ethanolic leaf extract of *O. stamineus* has the potential to be a novel treatment for epileptic seizures. To that end, a toxicity study was carried out to determine if the extract is safe for use with zebrafish, as well as to determine the doses to be used for the following behavioral study. The toxicity study had to be conducted as no prior published study using this extract has been conducted on adult zebrafish before. A prior literature search only yielded *O. stamineus* toxicity studies on Sprague Dawley rats (Chin et al., 2008) and zebrafish embryos (Ismail et al., 2017), and thus this work represents the first of its kind. The reason that an *O. stamineus* ethanolic extract was used is because ethanolic extracts of *O. stamineus* tend to have the highest concentration of phenolic compounds, followed by methanolic and aqueous extracts (Saidan et al., 2015). Thus, as oxidative stress plays a role in epilepsy (Devi et al., 2008) and that the phenols in *O. stamineus* such as rosmarinic acid possess significant free radical scavenging, anti-inflammatory and antioxidant ability (Akowuah et al., 2005; Yam et al., 2010), an ethanolic extract of *O. stamineus* is the ideal choice for this experiment. The results of Saidan et al. (2015) support this idea as they found that an ethanolic leaf extract of *O. stamineus* possess the greatest anti-oxidant activity from among a combination of ethanolic, methanolic and aqueous extracts. The reason that a leaf extract of *O. stamineus* was used over another part of the plant was due to experimental evidence such as that by Saidan et al. (2015) showing that extracts of the leaves possess anti-oxidant activity and that the traditional remedy for epilepsy utilizes the leaves of the plant (Hossain and Mizanur Rahman, 2015). Given the uncertainty associated with any novel experiment, the toxicity study used in this experiment follows a modified version of the OECD Guidelines for the Testing of Chemicals No. 203, which concerns acute toxicity tests in fish. The test involves the use of the test substance at a concentration of 100 mg/L of water, with a minimum of seven fish each for the treatment and control groups. The principle behind the test is that when there are no fish deaths after an exposure period of 96 h, the LC_50_ for the test substance can be said to be above 100 mg/L with a confidence of 99% or greater (OECD, 1992). As there have been no prior publications regarding the testing of the anti-convulsive potential of an ethanolic extract of *O. stamineus* in any animal species, this dose determination study was a necessity.

From the zebrafish swimming pattern after exposure to 200 and 400 mg/L of the *O. stamineus* ethanolic leaf extract, no bottom dwelling behavior was observed. Bottom dwelling in zebrafish in associated with anxiety and is initially seen in zebrafish which have just been transferred into a novel tank (Blaser and Rosemberg, 2012). As anxiolytics have been found to reduce bottom dwelling (Gebauer et al., 2011), the results of this study suggest that the extract has anxiolytic properties, at least at a concentration greater than 200 mg/L. The finding of this study that an overnight exposure to *O. stamineus* ethanolic leaf extract at a concentration of 400 mg/L is lethal to adult zebrafish is also noteworthy. This is because an acute oral toxicity study was performed using Sprague Dawley rats by Chin et al. (2008), by administering *O. stamineus* leaf extract up to a dose of 5.0 g/kg of rat body weight, daily for 14 days. The study by Chin et al. (2008) resulted in no rat deaths or any adverse effect on parameters such as body weight and they deemed that their methanolic *O. stamineus* whole plant extract seemingly lacked any toxic effects. Another toxicity experiment by Ismail et al. (2017) found that an aqueous extract of *O. stamineus* only significantly causes mortality in zebrafish embryos when the concentration reaches 5.0 g/L of water. However, both these experiments relied on a different manner of producing *O. stamineus* extracts compared to this study and thus may have a different proportion of constituents than the extract we used. In addition, a reliable correlation between zebrafish and rodent toxicities has not been established (Ducharme et al., 2015) and embryonic zebrafish toxicity may also not entirely correlate to adult zebrafish toxicity. Also, unlike dosing a rodent via the oral route, introducing a substance directly into the tank water makes it difficult to determine exactly how much of the substance has been taken up by the zebrafish (Kinkel et al., 2010). Thus, further work needs to be done to determine the mechanism behind the toxicity of *O. stamineus* ethanolic leaf extract in adult zebrafish to help reconcile the difference in toxicity results between this study and previous ones. It should also be noted that there is also no conversion factor for translating zebrafish toxicity to mammalian toxicity, although LC50 value zebrafish is generally lower than that for the corresponding rodent LC50 for certain chemicals such as polychlorinated biphenyls (Parng et al., 2002; Ducharme et al., 2015). However, given the popularity of *O. stamineus* as a traditional remedy for a plethora of illnesses, combined with multiple pharmacological studies demonstrating beneficial properties such as being hepatoprotective, antioxidant and antihypertensive (Ameer et al., 2012) as well as a relatively high toxic dose in rats (Chin et al., 2008), it is possible that *O. stamineus* derived AEDs would be relatively safe and non-toxic to humans.

Building on the toxicity study results, this experiment has also demonstrated that pre-treating zebrafish with an ethanolic leaf extract of *O. stamineus* for 30 min significantly increases the mean seizure onset time and decreases the mean seizure score of PTZ challenged zebrafish in a dose dependent manner. A 100 mg/L dose of the extract has been found in this study to rival the anti-convulsive effects of a 10 mg/L dose of the standard AED diazepam and a 200 mg/L dose of the extract has a stronger anti-convulsive effect than diazepam. The representative zebrafish swimming patterns also showed that diazepam reverses the bottom dwelling seen in PTZ challenge zebrafish, which is said to be comparable to the stupor like behavior and anxiety associated with an epileptic condition (Kundap et al., 2017). The swimming pattern produced by the zebrafish pre-treated with diazepam could be due to the sedative effect of diazepam, as it is a benzodiazepine (Gupta et al., 2014). In contrast to diazepam, zebrafish pre-treated with the extract produced a swimming pattern very similar to that of the vehicle control which was not challenged with PTZ. However, the 50 and 200 mg/L extract doses still produced some degree of bottom dwelling, although to a lesser degree than the negative control. This suggests that the 50 and 200 mg/L dose was insufficient to completely prevent the PTZ-induced seizures and this is supported by the mean seizure score for those doses being greater than zero. Interestingly, the 100 mg/L extract dose completely abolished bottom dwelling, although there was an increase in time spent on the water surface instead and the mean seizure score for 100 mg/L was also greater than zero. Taken together, the behavioral study results show that the *O. stamineus* ethanolic leaf extract does indeed possess dose dependent anti-convulsive properties but does not seem to produce the cognitive impairment associated with currently available AEDs such as diazepam.

Thus, our study shows that an *O. stamineus* ethanolic leaf extract derived novel AED has the potential to be comparable to diazepam, which is one of the standard AEDs available today. Undoubtedly, further work needs to be conducted to discover the active constituent/s of *O. stamineus* which contribute to its anti-convulsive properties. A follow up study similar to this one should then be conducted to test if a dose of the active constituent comparable to that of standard AEDs will still have similar or even better anti-convulsive efficacy. This is because our experiment shows that a dose of crude *O. stamineus* ethanolic leaf extract needs to be 10-fold that of diazepam to equal its effects. This is undesirable as high doses of substances in general tend to result in more side effects. Among the possible constituents responsible for the anti-convulsive effect an ethanolic leaf extract of *O. stamineus* are rosmarinic acid, sinensetin, eupatorine and 30-hydroxy-5,6,7,40-tetramethoxyflavone as they represent the major compounds in the extract which have anti-inflammatory action as well as substantial free radical scavenging and antioxidant ability (Pietta et al., 1991; Akowuah et al., 2005; Yam et al., 2010), all of which are factors that seem to protect against epilepsy (Devi et al., 2008; Vezzani et al., 2011). However, rosmarinic acid seems to be a likely candidate as several studies have found that it possesses anti-convulsive properties, possibly due to its activation of the GABAergic system (Khamse et al., 2015; Grigoletto et al., 2016) and hence promotion of inhibitory neurotransmission. Rosmarinic acid is also neuroprotective as a result of its anti-oxidant and free radical scavenging abilities (Fallarini et al., 2009). Data provided by the manufacturer of our standardized extract also reiterates the importance of rosmarinic acid as they found that rosmarinic acid (5.02%) was the most abundant of the four marker compounds they tested, followed by sinensetin (0.21%), eupatorine (0.17%), and 30-hydroxy-5,6,7,40-tetramethoxyflavone (Trace Amounts). Interestingly, doubling the dose from 50 to 100 mg/L produced a much larger positive effect on both mean seizure onset time and seizure score as compared to doubling the dose from 100 to 200 mg/L. This suggests that some yet unknown factor could be limiting the bioavailability of the extract, at least for the given exposure period of 30 min. However, it is worth reiterating that the actual amount of substance taken up by the zebrafish is not known when the substance is dissolved in the tank water, unlike methods such as an intraperitoneal injection whereby the quantity delivered is defined based on the weight of the fish (Kinkel et al., 2010). Despite the limitations of dissolving the *O. stamineus* ethanolic leaf extract directly into the tank water, it is utilized by this study as the AEDs used today for the chronic symptomatic treatment of epilepsy are given orally (Anderson and Saneto, 2012). Thus, as we are aiming to develop a novel AED based on an ethanolic *O. stamineus* leaf extract, it must also work through the oral route. This is because if the AED must be injected into a patient to work, it will likely be underutilized due to the chronic nature of epilepsy; regardless of its efficacy.

Based on the results of the gene expression study, the downregulation of NF-κB by the *O. stamineus* ethanolic leaf extract is unusual as inhibition of the NF-κB pathway usually results in a decreased seizure threshold (Yu et al., 1999). This could be explained by the extract controlling the PTZ-induced seizures via another mechanism and hence there is minimal activation of the NF-κB pathway. This theory is supported by the fact that diazepam also reduces the NF-κB expression level in comparison to the negative control and that the CP, T100, and T200 groups displayed a NF-κB expression level very similar to that of the baseline expression level in the CV group. As NF-κB also regulates the expression level of BDNF during seizures (Lubin et al., 2007), the BDNF expression levels should also mirror that of NF-κB. However, we found no significant upregulation in the BDNF expression level after a PTZ-induced seizure for any pre-treated group as compared to the negative control. However, the role of BDNF in the development of seizures and epilepsy is somewhat controversial as although there is usually an upregulation of BDNF is associated with a seizure, it is unclear whether this promotes or inhibits seizure development (Lubin et al., 2007). In the case of NPY, our results are unusual, with diazepam and the 50 mg/L extract dose not having a significant effect on the NPY expression level as compared to the negative control whereas the 100 and 200 mg/L dose decreased it to around the same as the baseline vehicle control level. Although only the 100 mg/L group represented a significant change, the unusual results could be explained due to the anti-convulsive effect of NPY and also its regulation of learning and memory (Colmers and El Bahh, 2003). The 50 mg/L still produced an upregulation in NPY as it does not sufficiently control the PTZ-induced seizures on its own and thus requires the assistance of NPY. Whilst diazepam does control the PTZ-induced seizures, it also negatively affects cognitive abilities (Kundap et al., 2017) and hence an upregulation of NPY is needed to counteract the cognitive dysfunction which results. The explanation for the decrease in the expression level of NPY for the 100 and 200 mg/L treatment groups is similar to that of NF-κB, as the seizures are controlled via other mechanisms and thus the NPY expression level is similar to the baseline vehicle control.

In the case of c-Fos expression, we found no significant upregulation as a result of a PTZ-induced seizure and no significant difference in c-Fos expression levels as a result of any treatment. However, according to literature, a seizure usually results in an increase in c-Fos expression (Peng and Houser, 2005). This discrepancy could be explained by the time between the PTZ challenge and removal of the zebrafish brain, which was 10 min in our experiment. According to Barros et al. (2015), in the case of rodents at least, c-Fos takes around 30 min to become significantly elevated from baselines levels after challenging with a pro-convulsant. It is possible that in our experiment, there was not enough time for c-Fos expression to become significantly elevated. For TNF-α, we found that there was a significant increase in TNF-α expression as a result of a PTZ-induced seizure, which is consistent with the results found in literature (Wilcox and Vezzani, 2014). Although all pre-treatments significantly decreased the TNF-α expression level, the T100 and T200 groups had a slightly lower expression level than the baseline vehicle control. This suggests that the ethanolic *O. stamineus* leaf extract may at least partially exert its anti-convulsive effect by acting as an anti-inflammatory agent as TNF-α is involved in systemic inflammation. The anti-inflammatory action of the extract may in turn be due to the downregulation of TNF-α by the extract, along with IL-1, COX-1 and COX-2 as determined by Tabana et al. (2016). The last gene we tested was IL-1, which was found to have no significant upregulation in the expression level after a PTZ-induced seizure, nor any other significant change for any pre-treated group as compared to the negative control. Whilst this contrasts with reports in literature about an increase in IL-1 levels after a seizure and the ability of the extract to decrease IL-1 expression levels (Tabana et al., 2016), there are conflicting reports which describe a decrease in IL-1 levels after a seizure (Rijkers et al., 2009). The role of IL-1 in seizures also currently remains unknown and controversial (Rijkers et al., 2009).

## Future Directions

Whilst this work represents a significant step in bridging the research gap, further research needs to be conducted on the discovery of the active anti-convulsive compound in the extract. Once identified, dose comparison studies with currently available AEDs should be conducted for a true test of their relative efficacies. Another area of future research is the usage of zebrafish tests such as the T-maze, which is design to assess the cognitive ability of the zebrafish (Stewart and Kalueff, 2012). This would help to determine if the extract does not cause cognitive impairment in zebrafish as our zebrafish swimming pattern results suggest.

## Conclusion

In conclusion, an ethanolic leaf extract of *O. stamineus* has the potential to be a novel symptomatic treatment for epileptic seizures as it is pharmacologically active against seizures in a zebrafish model. The anti-convulsive effect of this extract is also comparable to that of diazepam at higher doses and can surpass diazepam in certain cases. Treatment with the extract also counteracts the upregulation of NF-κB, NPY, and TNF-α as a result of a PTZ treated seizure. The anti-convulsive action for this extract could be at least partially due to its anti-inflammatory effects due to the downregulation of TNF-α.

## Ethics Statement

The experimental protocol was approved by the Monash Animal Research Platform (MARP) Animal Ethics Committee, Monash University, Australia (MARP/2017/047).

## Author Contributions

BC performed all the experiments and was responsible for the writing of the manuscript in its entirety. UK performed the gene expression study in tandem with BC. MS was responsible for conceptualizing and revising the manuscript. YK, S-MH, and IO were also involved in conceptualizing and proofreading. All authors gave their final approval for the submission of the manuscript.

## Conflict of Interest Statement

The authors declare that the research was conducted in the absence of any commercial or financial relationships that could be construed as a potential conflict of interest.
